# Current surgical management for melanoma

**DOI:** 10.1111/1346-8138.17086

**Published:** 2023-12-27

**Authors:** Shigeru Koizumi, Takashi Inozume, Yasuhiro Nakamura

**Affiliations:** ^1^ Department of Skin Oncology/Dermatology Saitama Medical University International Medical Center Saitama Japan; ^2^ Department of Dermatology Chiba University Chiba Japan

**Keywords:** malignant melanoma, neoadjuvant therapy, regional lymph node dissection, sentinel lymph node biopsy, surgery

## Abstract

Melanoma is a major malignant cutaneous neoplasm with a high mortality rate. In recent years, the treatment of melanoma has developed dramatically with the invention of new therapeutic agents, including immune checkpoint inhibitors and molecular‐targeted agents. These agents are available as adjuvant therapies for postoperative patients with stage IIB, IIC, and III melanomas. Furthermore, neoadjuvant therapy has been studied in several global clinical trials and has demonstrated promising and favorable clinical efficacy, mainly in patients with palpable regional lymph nodes. A recent large phase III clinical trial investigating early lymph node dissection for sentinel lymph node metastases demonstrated no survival benefits. Based on these data, surgery should be reconsidered as an appropriate treatment modality for melanoma. The need for invasive surgical procedures will be reduced with the invention of effective adjuvant and neoadjuvant therapies and novel clinical trial data on regional lymph node dissection. However, surgery still plays an important role in treating early‐stage melanoma, accurately determining the disease stage, and effective palliative treatment for advanced melanoma. In this article, we focus on surgery for primary tumors, regional lymph nodes, and metastatic sites in an era of remarkably revolutionary drug treatments for melanoma.

## 1 INTRODUCTION

Melanoma has a high mortality rate and is one of the most aggressive malignant cutaneous neoplasms. The incidence of melanoma is increasing worldwide.^1^, ^2^ According to the Global Cancer Observatory (GLOBOCAN) series of the International Agency for Research on Cancer, the global estimated age‐standardized rates per 100 000 person‐years of cutaneous melanoma incidence were 3.3 in male and 2.8 in female in 2012.^1^ In 2018, the estimated age‐standardized rates were 3.5 in male and 2.9 in female.^2^

Melanoma is considered a multifactorial disease arising from the interaction between environmental exposure and genetic susceptibility.^3^ The most important and preventable environmental risk factor is exposure to ultraviolet (UV) light. UV radiation can cause the formation of oxidized melanin, forming various radicals and disrupting reactive oxygen species, which results in melanoma.^4^ The major host risk factors are family history and genetic susceptibility.^3^ Mutations in cyclin‐dependent kinase inhibitor 2A (*CDKN2A*) and p16 are often observed in these patients.^5^

Surgery was the gold standard of care for resectable melanoma owing to the lack of effective therapeutic agents for melanoma before the era of the development of novel therapeutic agents, such as immune checkpoint inhibitors (programmed death 1 [PD‐1] and cytotoxic T‐lymphocyte‐associated protein 4 [CTLA‐4] inhibitors) and molecular‐targeted agents (BRAF inhibitors and MEK inhibitors). After the invention of those agents, the prognosis of resected high‐risk melanoma (stage IIB–IIC, stage III, and stage IV melanoma) improved by the use of these adjuvant therapy agents.^6^, ^7^, ^8^, ^9^ Several phase II clinical trials investigating the efficacy of neoadjuvant therapies using PD‐1 inhibitor with or without CTLA‐4 inhibitor or BRAF inhibitor plus MEK inhibitor have demonstrated promising favorable clinical efficacy mainly in patients with palpable regional lymph nodes, but these treatments are not yet approved worldwide.^10^, ^11^, ^12^, ^13^, ^14^, ^15^, ^16^, ^17^, ^18^ Additionally, a recent large phase III clinical trial, the Multicenter Selective Lymphadenectomy Trial II (MSLT‐II), investigating the role of early regional completion lymph node dissection (CLND) for sentinel lymph node (SLN) metastasis showed no superior survival benefit of early CLND compared with observation cohort.^19^ The invention of effective adjuvant and neoadjuvant therapies and novel clinical trial data regarding CLND will lead to less‐invasive surgery in melanoma treatment. Although a new standard of care involving less‐invasive surgical treatment combined with novel agents will be developed, surgery still plays an important role in treating early‐stage melanoma, accurately determining the disease stage and effective palliative treatment for advanced melanoma.

## 2 WIDE LOCAL EXCISION FOR PRIMARY TUMOR

In resectable locoregional melanomas, surgery for primary tumors aims to excise the tumor completely. The side margins of wide local excision, a fundamental surgical procedure, have historically changed in several clinical trials.

### 2.1 Side margins

In the past, wide‐sided margin excision was thought to improve local control and long‐term prognosis. Historical surgical approaches for primary tumors have been aggressive, and wide local excision with a side margin of 5 cm is commonly performed.^20^

Six randomized controlled trials have been conducted to compare wider and narrower margins for patients with various Breslow tumor thicknesses (TTs) (Table 1).^21^, ^22^, ^23^, ^24^, ^25^, ^26^, ^27^ A first randomized trial reported in 1991 researched the relationship between the different side margins (1 cm vs ≥ 3 cm), local recurrence, and overall survival (OS) in 612 patients with a TT of 0.8–2.0 mm. The local recurrence rates during follow‐up (median, 7.5 years) were similar between the two groups (1.3% vs 0%). OS showed no statistical significance (8‐year OS: 86.9% and 90.3%, *P* = 0.64).^21^, ^28^, ^29^ Another randomized trial^22^ reported in 2000 analyzed the relationship between different side margins (2 cm vs 5 cm) and local recurrence, relapse‐free survival (RFS), and OS rates in 989 patients with a TT of 0.8–2.0 mm. During a median follow‐up of 11 years, local recurrence rates were similar between the two groups (0.6% vs 1%). No statistical significance was found in RFS and OS (10‐year RFS 71% vs 70%, 10‐year OS 79% vs 76%, *P* values unavailable).^22^ Another randomized trial^23^ reported in 2001 compared local recurrence and OS between two different side margins (2 cm vs 4 cm) excised in 468 patients with a TT of 1.0–4.0 mm. Following a median follow‐up of 10 years, there was no statistical significance in local recurrence and OS between the two groups (local recurrence rates 2.1% vs 2.6%, *P* value unavailable; 10‐year OS 70% and 77%, *P* = 0.074).^23^ Furthermore, a randomized multicenter trial^24^ reported in 2003 researched local recurrence, disease‐free survival (DFS), and OS in the two different margins (2 cm vs 5 cm) in 326 patients with a TT of ≤2 mm. The local recurrence rates were similar between the two groups (0.6% vs 2.4%). No statistical significance was observed in DFS and OS (10‐year DFS 85% vs 83%, *P* = 0.83; 10‐year OS 87% vs 86%, *P* = 0.56).^24^ Moreover, another randomized multicenter trial^25^ reported in 2011 analyzed local recurrence and OS comparing two different margins (2 cm vs 4 cm) in 936 patients with thickness >2 mm. No statistically significant difference between the two groups was observed in the local recurrence rates (4.3% vs 1.9%, *P* = 0.06). RFS and OS also had no statistical significance (5‐year RFS 56% vs 56%, *P* = 0.69; 5‐year OS 65% and 65%, *P* = 0.69).^25^ Lastly, a randomized multicenter trial^26^, ^27^ reported in 2004 and 2016 compared local recurrence, DFS, and OS in the different excisional margins (1 cm vs 3 cm) in 900 patients with a TT >2.0 mm. There was a statistically significant difference in the locoregional recurrence (local recurrence, in‐transit metastasis, and regional lymph node metastasis) rate between the two groups (37.0% vs 31.8%, *P* = 0.05). Statistically significant differences were found in melanoma‐specific survival (MSS) (5‐year MSS 57.1% vs 63.6%, *P* = 0.04). However, there was no statistically significant difference in OS (44.1% vs 46.8% *P* = 0.14).^26^, ^27^ Based on the results of the randomized trials above (Table 1), the current National Comprehensive Cancer Network (NCCN) Guidelines^30^ recommend side margins according to Breslow thickness, as shown in Table 2.

**TABLE 1:** Studies evaluating different surgical side margins for wide local excision.

Author	Published year	Total sample size	Median or mean follow‐up period (years)	Tumor thickness (mm)	Side margin (cm)	Local recurrence	Overall survival
Veronesi et al.^28^, ^29^	1991	612	7.5	≤2.0	1 vs. ≥3	Similar	Similar
Cohn‐Cedermark et al.^22^	2000	989	11	0.8–2.0	2 vs. 5	Similar	Not significant
Balch et al.^23^	2001	468	10	1.0–4.0	2 vs. 4	Not significant	Not significant
Khayat et al.^24^	2003	326	16	≤2.0	2 vs. 5	Similar	Not significant
Gillgren et al.^25^	2011	936	6.7	>2.0	2 vs. 4	Not significant	Not significant
Thomas et al.^26^ Hayes et al.^27^	2004 2016	900	8.8	>2.0	1 vs. 3	Significant^a^	Not significant^b^

^a^ Including local recurrence, in‐transit metastasis, and regional lymph node metastasis.

^b^ Statistically significant in melanoma‐specific survival.

**TABLE 2:** Recommended surgical side margins according to tumor thickness in the National Comprehensive Cancer Network Guidelines.

Tumor thickness (mm)	Recommended side margin (cm)
In situ	0.5–1
≤1.0	1
1.01–2.0	1–2
≥2.01	2

However, all these clinical trials targeted Caucasian patients with melanoma mainly occurring in the trunk and extremities. Very few patients with acral melanoma were included in these trials. The appropriate side margins for these rare melanoma clinical subtypes have not been investigated. Recently, several studies focusing on the side margins of acral melanoma have been reported.^31^, ^32^, ^33^ A retrospective study^31^ including 100 Japanese patients with invasive acral melanomas compared clinical outcomes in patients treated with margins recommended by the NCCN Guidelines^30^ (R group) with those treated with narrower margins (N group). In patients with T1‐3 melanoma, the mortality rates in the N and R groups were almost identical (1.36 and 1.28 per 100 person‐years). Among patients with T4 melanoma, the N group had a higher mortality rate than the R group (11.44 vs 5.03 per 100 person‐years). Multivariate analysis showed that surgical margin was not a risk factor for MSS (*P* = 0.38) or DFS (*P* = 0.31).^31^ Additionally, Lino‐Silva et al.^32^ analyzed recurrence (local recurrence or distant metastasis) rates and OS between two margins (1–2 cm vs > 2 cm) in 306 patients with acral melanoma at pathologic stages pT3 and pT4. No statistical significance was found in recurrence rates and OS between the two groups (mean follow‐up of 31.9 months, recurrence rates 30.0% vs 35.4%, *P* = 0.32; 5‐year OS 69.9% vs 61.4%, *P* = 0.41).^32^ Sun et al.^33^ compared local and in‐transit recurrence‐free survival (LITRFS), DFS, OS in the different side margin excisions (1–2 cm vs > 2 cm) in 207 patients with acral melanoma with stage pT3‐T4 disease. The side margin was not correlated with patients' LITRFS, RFS, or OS (LTTRF, *P* = 0.35; DFS, *P* = 0.08; OS, *P* = 0.20).^33^

As for an ongoing trial, the Melanoma Margins Trial II (MelMarT‐II; ClinicalTrials.gov number, NCT03860883) compared clinical outcomes between 1‐cm margin wide local excision and 2‐cm margin wide local excision in patients with a Breslow thickness of >2 mm or 1–2 mm with ulceration (pT2b‐pT4b).

### 2.2 Deep margins

The appropriate determination of deep margins is as crucial as that of side margins for complete removal of the primary tumor. However, there is a lack of studies investigating adequate deep margins.^34^, ^35^ The NCCN Guidelines do not clearly state recommendations for deep margins.^30^ A retrospective study by Grotz et al.^35^ compared the clinical outcomes of different deep margins between the fascia resection and preserved groups. Fascial resection was associated with a 2.5‐fold increased risk of regional nodal recurrence. However, it was not associated with local recurrence or OS,^35^ indicating that wide local excision with underlying fascia resection would not improve local control and survival. Additionally, this study had a considerable selection bias, as the TT was thicker in the fascial resection group. In general, the distance from the deepest base of the primary tumor to the fascia varied greatly, depending on the patient's obesity. Therefore, in real‐world practice, the determination of deep margins appears to depend on the discretion of the individual surgeon.

**FIGURE 1:**
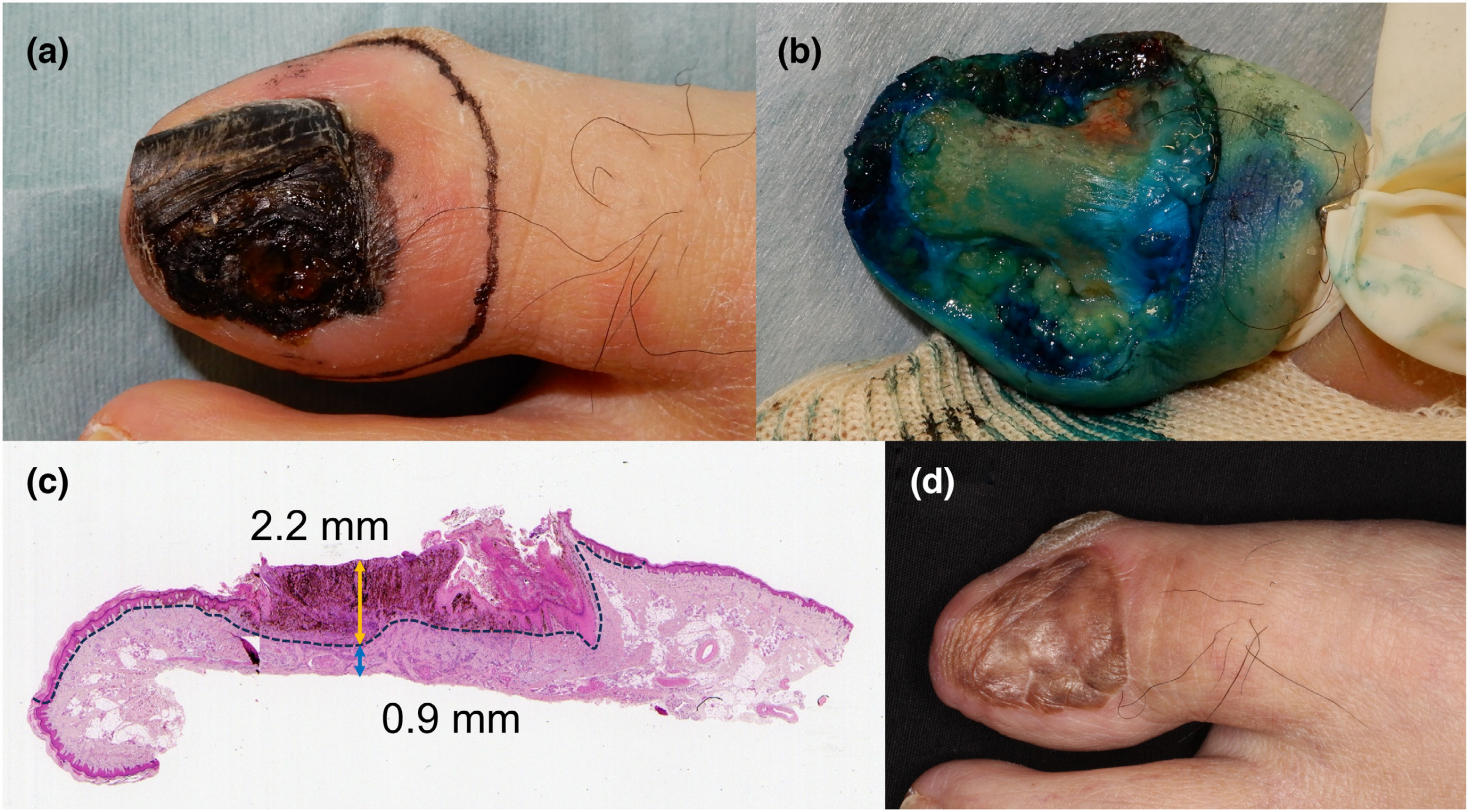
A male patient with invasive nail apparatus melanoma who was enrolled in JCOG 1602 and received non‐amputative digit preservation surgery. (a) Black‐colored nail plate with ulcerative nodular lesion and Hutchinson's phenomenon on the left big toenail. Black line indicates incision line of non‐amputative digit preservation surgery. (b) After removal of the nail apparatus at the level of the distal phalanx. (c) Histopathology of resected nail apparatus. The black dotted line indicates the proliferative area of melanoma cells. The yellow double‐ended arrow indicates tumor thickness (2.2 mm). The blue double‐ended arrow indicates the shortest tumor‐to‐deep margin distance (0.9 mm). (d) Clinical appearance 3 years after coverage of defect by skin grafting.

Studies on appropriate deep margins in nail apparatus melanoma are crucial for preserving digit function. Although non‐amputative digit preservation surgery is applied to in situ or <0.8‐mm TT nail apparatus melanoma,^30^ amputation surgery has been the standard of care due to the proximity of the nail matrix to the distal phalanx.^37^, ^38^, ^39^ The loss of a digit by amputation leads to poor cosmesis and interferes with activities of daily living. Meanwhile, the prognosis in patients with nail apparatus melanoma depends on the T stage at the initial diagnosis, not the degree of radical surgery for the primary tumor.^36^ Several case reports or series of patients with invasive nail apparatus melanoma treated with non‐amputative digit preservation surgery, with 5–10 mm side margins, found a low recurrence incidence and favorable survival.^40^, ^41^, ^42^, ^43^ A recent histologic study also supported that many patients with nail apparatus melanoma with TT of ≤4 mm did not show invasion to the underlying distal phalanx.^44^ Based on the results from these studies, non‐amputative digit preservation surgery may be applicable even in patients with invasive nail apparatus melanoma without compromising their digit cosmesis, digit function, and vital prognosis. An investigator‐initiated clinical trial, JCOG1602 (J‐NAIL study, clinical trial no. UMIN000029997), to evaluate the safety and efficacy of non‐amputative digit preservation surgery with a side margin of 5–10 mm in patients with invasive nail apparatus melanoma is ongoing in Japan (Figure 1).^45^

## 3 SLN BIOPSY

The SLNs are the first regional lymph nodes to receive lymphatic flow from a primary tumor. If the SLNs do not metastasize, it is considered that there are no regional metastatic nodes downstream of the SLNs. In this context, SLN biopsy (SLNB) remains an important diagnostic procedure for detecting nodal micrometastases and predicting the prognosis.

### 3.1 Development of SLNB techniques

To identify the SLN, blue dye injection around the primary tumor was performed in the first advent of the SLNB concept. However, the SLN identification rate with blue dye injection alone was low, approximately 82%.^46^ Lymphoscintigraphy using radioisotope (RI) injection, such as technetium tin colloid or technetium phytate, and the intraoperative use of a hand‐held gamma probe (RI method) dramatically increased the SLN identification rate. Combined with the RI method, blue dye injection significantly improved SLN identification by up to 99%,^47^, ^48^ and has been the standard technique for SLNB. Additionally, hybrid single‐photon emission computed tomography/computed tomography (SPECT/CT) can accurately visualize the anatomic position of radioactive SLNs and interval nodes, which is of great value when radioactive SLNs are located in complex anatomical areas such as the head and neck region.

Furthermore, the development of intraoperative indocyanine green (ICG) fluorescence imaging (the ICG method) in SLNB has demonstrated higher SLN identification rates.^49^, ^50^, ^51^, ^52^, ^53^ ICG binds to albumin in the human body after injection and generates a peak wavelength of 840 nm near‐infrared fluorescence when excited with 765 nm light. The intraoperative use of a near‐infrared camera enabled dermatologic surgeons to observe ICG as superficial subcutaneous lymphatic flow. The small particle size of ICG (2.1 nm) also led to the smooth flow of ICG in the lymphatics and easy identification of SLNs that were not identified by lymphoscintigraphy because of the poor flow of the RI due to its larger particle size than ICG. The synergistic use of the ICG method with RI method has been proposed in cases of difficult identification of SLN using the RI method alone, such as the shine‐through phenomenon due to the proximity between the primary tumor and radioactive SLNs.^54^

### 3.2 Complications of SLNB

The complications induced by SLNB vary among reported studies.^55^, ^56^, ^57^ A systematic review reported in 2017^57^ analyzed 9047 patients in 21 articles, and the overall incidence rate of complications was 11.3%. Seroma was the most common complication (5.1%), followed by infection (2.9%), hematoma (0.5%), lymphedema (1.3%), and nerve injury (0.3%). No significant differences were found in the incidence of complications among the SLNBs in the different lymphatic basins.

### 3.3 Target patients for SLNB

SLNB is a crucial procedure for the accurate pathological staging of patients with clinical stages I and II. SLNB is generally recommended for patients with a TT of ≥0.8 mm because the probability of harboring positive SLNs in patients with a TT of <0.8 mm without ulceration (T1a disease) is extremely low (<5%).^58^ Additionally, a recent retrospective study^59^ in patients with T1a disease demonstrated that significant high‐risk factors for positive SLN are associated with age ≤42 years (7.5%), head/neck primary tumor (9.2%), lymphovascular invasion (21.4%), and ≥2 mitoses/mm^2^ (8.2%). When patients had two factors of age <42 years and ≥2 mitoses/mm^2^, the rate of positive SLN was 18.4%.^59^

Based on the above recent data, current NCCN Guidelines^30^ indicate that SLNB should be discussed and considered if the patient has T1b disease (TT of <0.8 mm with ulceration or 0.8–1 mm with or without ulceration) or T1a disease with a TT of ≥0.5 mm with other adverse features of age ≤42 years, head/neck primary tumor, lymphovascular invasion, and/or mitotic index ≥2/mm^2^.

### 3.4 Management of patients with positive SLN

Although CLND has traditionally been performed in patients with positive SLN, the results of recent pivotal phase III randomized trials investigating the therapeutic value of immediate CLND after positive SLNB and the advent of novel adjuvant therapies, including immune checkpoint inhibitors and BRAF/MEK inhibitors, have changed this traditional strategy.

As a first phase III randomized clinical trial, the Dermatologic Cooperative Oncology Group‐SLN Trial (DeCOG‐SLT)^60^ was conducted to investigate the therapeutic role of immediate CLND in patients with a TT of ≥1.0 mm melanoma harboring positive SLN. Comparing the immediate CLND cohort (*n* = 240) with the observation cohort (*n* = 233, patients who underwent CLND when regional lymph node metastasis was suspected based on ultrasonographic examination), no significant differences were observed in the distant metastasis‐free survival (DMFS) (5‐year DMFS 67.6% vs 64.9%, hazard ratio [HR] 1.08, *P* = 0.87), RFS (HR, 1.01), and OS (HR, 0.99) between the two treatment cohorts, but most patients (*n* = 311) had small tumor burdens of ≤1.0 mm in the SLNs. Another representative pivotal trial, MSLT‐II,^19^ enrolled a larger number of patients with positive SLN and compared the immediate CLND cohort (*n* = 824) with the observation cohort (*n* = 931). There was no significant difference in MSS between the two cohorts (mean 3‐year MSS rate 86 ± 1.3% vs 86 ± 1.2%, *P* = 0.42). The DFS was slightly prolonged in the CLND cohort but insignificant (3‐year DFS rate 68 ± 1.7% and 63 ± 1.7%, *P* = 0.05). Furthermore, the incidence of postoperative lymphedema was higher in the CLND cohort than in the observation cohort (24.1% vs 6.3%). A positive non‐SLN status, determined by CLND, was an independent prognostic factor for recurrence (HR 1.78, *P* = 0.005). Based on the data from the two major clinical trials mentioned above, immediate CLND was not associated with prolonged survival, although immediate CLND may provide patient prognostic information.

The advent of effective adjuvant therapies using novel agents, including anti‐PD‐1 antibodies and BRAF/MEK inhibitors, has drastically changed the real‐world management of patients with positive SLN. Simultaneous pivotal phase III clinical trials for adjuvant systemic therapy showed that anti‐PD‐1 antibody (nivolumab, pembrolizumab) and BRAF/MEK inhibitors are more effective than historical alternatives, such as high‐dose interferon‐alpha or observation with no adjuvant treatment. The protocols of these pivotal phase III clinical trials, including CheckMate 237,^61^ KEYNOTE‐054,^7^ and COMBI‐AD,^62^ investigating the efficacy of nivolumab, pembrolizumab, and dabrafenib plus trametinib combination as adjuvant therapies, all included patients who received adjuvant therapy after CLND, even if they had positive SLNs. However, in real‐world practice, physicians often administer adjuvant therapy after skipping CLND in patients with positive SLNs. A recent large retrospective study^63^ enrolled 1109 patients with positive SLN from 21 institutions in Australia, Europe, and the United States. The results showed that 47% of patients (*n* = 519) were treated with nodal observation alone, 37% of patients (*n* = 411) were treated with nodal observation plus adjuvant systemic therapy, 9% of patients (*n* = 102) were treated with CLND alone, and only 7% of patients (*n* = 77) received CLND plus adjuvant therapies.^63^ Another retrospective study^64^ from Japan also demonstrated that the proportion of patients with positive SLN who underwent CLND was significantly lower after the publication of the MSLT‐II trial results and approval of novel adjuvant therapies (66.1% [37/56] vs 90.5% [57/63], *P* = 0.001).

## 4 CLND

As CLND for patients with positive SLN tends to be omitted owing to recent evidence from DeCOG‐SLT and MSLT‐II, and novel effective adjuvant therapies, the current real‐world indication of CLND is considered for patients with clinical nodal disease. However, the true therapeutic value of CLND for clinical nodal disease is still unknown because no trials have compared the prognosis after CLND with that without CLND or alternative nonsurgical treatment modalities in those cohorts, therefore CLND provides regional control and prognostic information. The 5‐year OS after CLND ranges from 30% to 50%, depending on the number of metastatic lymph nodes, extracapsular extension, and high‐risk primary tumor features, such as TT, ulceration, and location.^65^, ^66^, ^67^, ^68^, ^69^, ^70^, ^71^, ^72^, ^73^

### 4.1 Complications of CLND

The rates and types of complications in CLND vary among different studies, lymphatic basins of CLND (neck, axillary, inguinal, pelvic, and popliteal), and the extent of CLND. Generally, the rates of complications in axillary and ilioinguinal CLND are reported to be between approximately 20% and 60%.^74^, ^75^, ^76^ Frequent complications include wound infection, wound breakdown, hematoma, seroma, neuropathy, lymphocele, and lymphedema.^66^, ^71^, ^74^, ^75^, ^76^, ^77^, ^78^, ^79^ Risk factors for CLND complications include obesity, old age, and inguinal CLND.^71^, ^76^, ^80^, ^81^ Lymphocele formation following inguinal CLND is frequently observed in clinical practice.^82^

### 4.2 Appropriate extent of CLND

The extent of CLND is often modified based on the anatomic location of the clinical nodal disease and lymphatic flow in the regional lymphatic basin. However, the appropriate extent of CLND that safely balances the therapeutic effects remains controversial. Although many studies have attempted to clarify the appropriate extent of CLND,^74^, ^83^, ^84^, ^85^, ^86^, ^87^ these studies included patients with positive SLN who no longer were absolute candidates for CLND.

#### 4.2.1 Neck CLND (neck dissection) and parotidectomy

**FIGURE 2:**
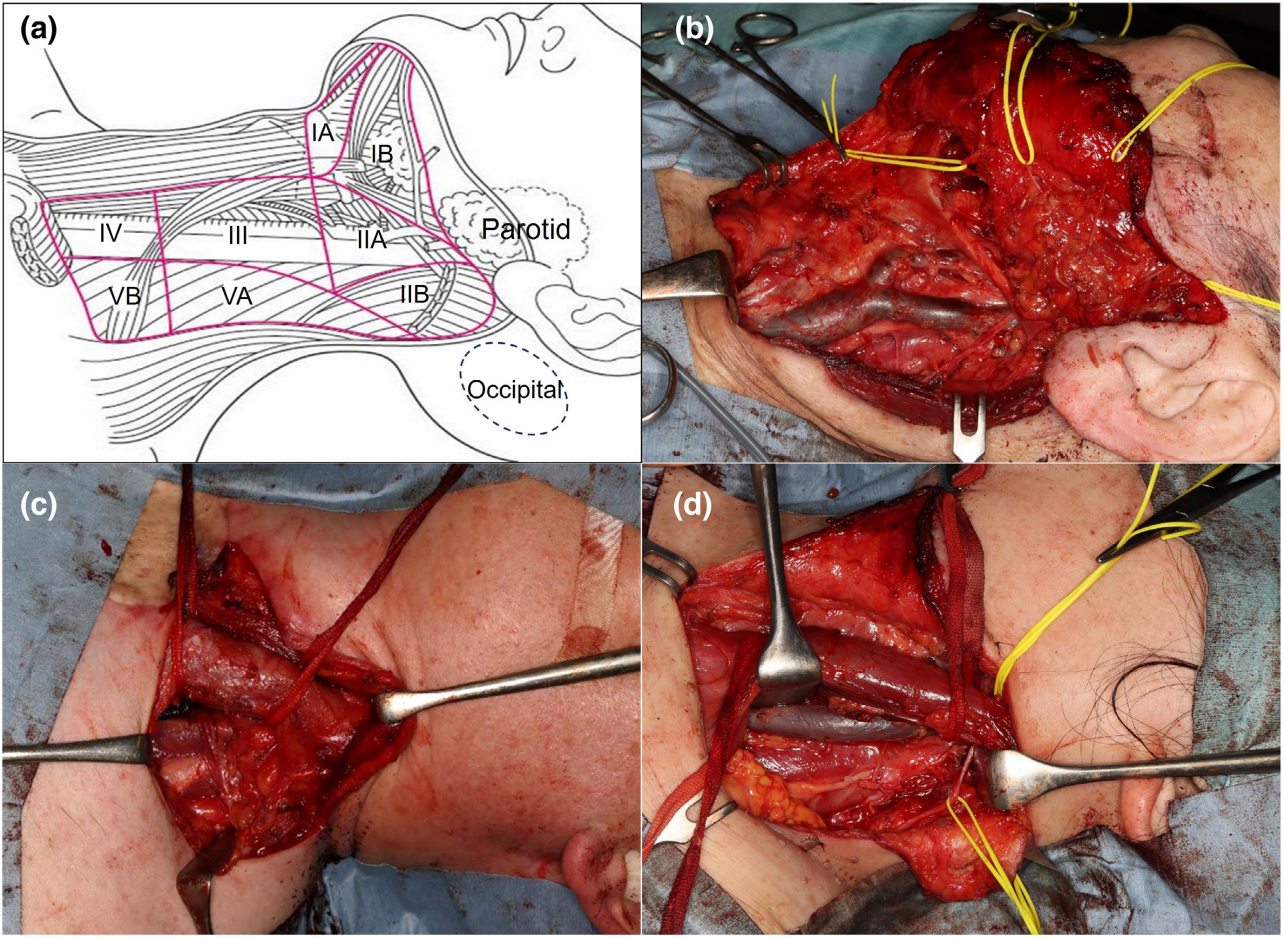
Different extents of selective neck dissection according to the location of primary tumor and clinical nodal disease. (a) Scheme of the dissection levels and areas in the left head and neck region. (b) A patient with melanoma on the left cheek receiving selective neck dissection (level IB, II, III, and superficial parotidectomy). (c) A patient with melanoma on the upper chest receiving narrow area selective neck dissection (level IV and VB). (d) A patient with melanoma on the left occipital region receiving selective neck dissection (level II–V and occipital nodes).

The lymphatic drainage pattern of the head and neck region is complicated, therefore selective neck dissection has become a major treatment of choice to minimize the extent of dissection.^88^ It depends on the predicted lymphatic flow from the location of the primary tumor and the different extent of dissection, including superficial parotidectomy,^89^ has been proposed by O'Brien et al.^90^ and the Netherland Cancer Institute^91^ as representative guides for selective neck dissection (Table 3 and Figure 2a–d). However, the extents of selective neck dissection of all primary tumors are not covered by those guides (Figure 2c) and there is no definitive evidence that the therapeutic effect of those extents of selective neck dissection is truly not inferior to that of a larger extent of dissection, including radical or modified radical neck dissection. Additionally, in the head and neck region, unlike head and neck cancer, dissection of superficial lymph nodes, such as preauricular, postauricular, occipital, and superficial cervical nodes, is sometimes required.^92^

**TABLE 3:** Recommended extent of neck dissection based on the predicted lymphatic drainage pattern from the location of the primary tumor to the cervical lymph node.

Location of primary tumor	Recommended extent of neck dissection	
	Proposed by O'Brien et al.^90^	Treatment protocol of the Netherlands Cancer Institute^91^
Anterior scalp	I–III or IV, P	I–V, P
Coronal scalp	I–V, P	I–V, P
Posterior scalp	I–V, O	II–V, O
Preauricular	I–III or IV, P	I–V, P
Ear	II–V, O	I–V, P
Upper face	I–III or IV, P	I–V, P
Nose	I–III or IV, P	I–V, P
Lower face	I–III or IV, P	I–V, P
Anterior upper neck	I–III or IV, P	I–V
Anterior lower neck	II–V	II–V
Coronal upper neck	I–V, P	I–V, P
Coronal lower neck	III–V	II–V
Posterior upper neck	II–V, O	II–V, O
Posterior lower neck	III–V	II–V, O

Abbreviations: O, occipital node; P, parotid node.

#### 4.2.2 Axillary CLND

The axial lymph nodes were divided into three levels (I, II, and III). Although the recommended level of CLND has been from I to III,^93^, ^94^, ^95^ no significant difference was observed in locoregional recurrence between the level I to III CLND group and the level I and II CLND group in patients with positive SLN.^93^ However, patients with positive SLN are no longer candidates for CLND. Further research is required to clarify the appropriate extent of axillary CLND in patients with nodal disease.

#### 4.2.3 Ilioinguinal CLND

The previous version of the NCCN Guidelines in 2020^96^ recommended consideration of ilioinguinal CLND if the PET/CT or pelvic CT scan shows the following: nodal involvement in the iliac and/or obturator nodes, a positive Cloquet's lymph node following intraoperative histologic evaluation, clinical nodal disease in the inguinal area, and three or more metastatic nodes. This strategy could be interpreted as all patients must consider ilioinguinal CLND if only the patients with clinical nodal disease proceed to CLND. In this respect, the Japanese Melanoma Guidelines published in 2019^97^ propose a different recommendation (“not to perform iliac and obturator CLND is suggested as an additional procedure in patients who need inguinal CLND”). This recommendation is based on the meta‐analysis of 10 studies involving the comparison of inguinal CLND with ilioinguinal CLND, which showed no statistical differences in the RFS or OS between the two treatment groups (RFS: odds ratio 1.00, 95% CI 0.90–1.10, *p* = 0.96; OS: odds ratio 1.06, 95% CI 0.95–1.17, *p* = 0.29). The Japanese Melanoma Guidelines panel members recommend considering ilioinguinal CLND in patients with radiologically evident metastases in both the pelvic and inguinal areas on radiologic imaging. Thereafter, the latest version of NCCN Guidelines^30^ modify recommendation of ilioinguinal CLND, which may be considered only if imaging shows resectable lymphadenopathy in pelvic areas.

## 5 WILL NEOADJUVANT THERAPIES LEAD TO LESS‐INVASIVE SURGERY?

As described above, the current standards of care for resectable clinical nodal disease (clinical stage III) are wide local excision and CLND, followed by adjuvant therapy. However, recent attempts, mainly in this clinical stage, have shown that these novel agents can be used as neoadjuvant therapies before surgery. Neoadjuvant therapies have the potential advantages of tumor reduction, resulting in less‐invasive excision for primary lesions, omission of CLND, and prolonged survival, but there may be a risk of disease progression during neoadjuvant therapies if ineffective.^98^ In general, several recent clinical trials of neoadjuvant therapies, mainly for patients with clinical stage III melanoma, have shown favorable clinical efficacies (Table 4).^10^, ^11^, ^12^, ^13^, ^14^, ^15^, ^16^, ^17^, ^18^ The latest representative phase II study, PRADO trial,^17^ using nivolumab plus ipilimumab combination as neoadjuvant therapies, showed a pathologic complete response (pCR) of 49% and near‐pathologic complete response of 12%, which led to the omission of CLND after neoadjuvant therapy. The 2‐year RFS in the entire cohort was 85% (2‐year RFS in patients with pCR + near pCR was 93%). The other representative phase II trial^18^ that used pembrolizumab as neoadjuvant therapy and adjuvant therapy compared with the adjuvant pembrolizumab alone, in patients with resectable stage IIIB to IV melanoma, also demonstrated favorable outcomes. The neoadjuvant‐adjuvant cohort (154 patients) revealed pCR of 21% and statistically significant prolonged event‐free survival compared to the adjuvant‐only cohort (159 patients) (2‐year event‐free survival 72% vs 49%, *P* = 0.004). The 2‐year OSs of each cohort were >80% and <80%, respectively (*P* value unavailable). These results suggest a potentially superior therapeutic effect of neoadjuvant therapy compared with adjuvant therapy and the possibility of a personalized surgical approach in combination with immune checkpoint inhibitors such as neoadjuvant therapy in these stages,^17^ which may be a new standard of care.

**TABLE 4:** Clinical trials for neoadjuvant therapy using immune checkpoint inhibitors or molecular‐targeted agents.

Author, year (trial name)	Stage	*N*	Treatment arm	Pathological complete response rate	RFS	OS
Amaria et al., 2018^10^	Resectable clinical stage III or oligometastatic stage IV	23	≤4 doses of NAT nivo (3 mg/kg) ≤3 doses of NAT ipi (3 mg/kg) + nivo (1 mg/kg)	25% (nivo) 45% (ipi + nivo)	80% at 20.5 months (nivo) 90% at 14.9 months (ipi + nivo)	76% at 22.6 months (nivo) 100% at 24.4 months (ipi + nivo)
Blank et al., 2018^11^ (OpACIN)	Palpable stage III	20	Adj arm: 4 doses of Adj ipi (3 mg/kg) + nivo (1 mg/kg) NAT → Adj arm: 2 doses of NAT ipi (3 mg/kg) + nivo (1 mg/kg) → 2 doses of Adj ipi (3 mg/kg) + nivo (1 mg/kg)	33% (NAT → Adj arm)	78% at 24 months (Adj arm) 80% at 24 months (NAT → Adj arm)	>70% at 24 months (Adj arm) >80% at 24 months (NAT → Adj arm)
Rozeman et al., 2019^12^ (OpACIN‐neo)	Resectable stage III involving lymph nodes only	89	Arm A: 2 doses of NAT ipi (3 mg/kg) + nivo (1 mg/kg) Arm B: 2 doses of NAT ipi (1 mg/kg) + nivo (3 mg/kg) Arm C: 2 doses of NAT ipi (3 mg/kg) → 2 doses of Adj nivo (3 mg/kg)	47% (Arm A) 57% (Arm B) 23% (Arm C)	<80% at 12 months (Arm A) >80% at 12 months (Arm B) >80% at 12 months (Arm C)	N/A
Huang et al., 2019^13^	Resectable stage IIIB/C or IV	27	1 dose of NAT pembro (200 mg) → 12‐month Adj pembro	19%	N/A	N/A
Long et al., 2019^14^	Clinical stage IIIB/C	35	3‐month NAT dab (300 mg) + tra (2 mg) → 10‐month Adj dab +tra	49%	>40% at 24 months	N/A
Blankenstein et al., 2021^15^ (NeoCombi)	Unresectable BRAF‐mutated locally advanced stage IIIC or oligometastatic stage IV	21	2‐month NAT dab (300 mg) + tra (2 mg)	29%	9.9 months in median	>70% at 60 months
Amaria et al., 2021^16^	Stage IIIB‐IV (M1a)	30	2 doses of NAT nivo (480 mg) + rela (160 mg) → 10 doses of Adj nivo + rela	59%	93% at 12 months	95% at 12 months
Reijers et al., 2022^17^ (PRADO)	Stage IIIB‐IIID	99	2 doses of NAT ipi (1 mg/kg) + nivo (3 mg/kg)	49%	85% at 24 months	N/A
Patel SP et al., 2023^18^	Stage IIIB‐IV	313	Adj arm: 18 doses of Adj pembro (200 mg) NAT → Adj arm: 3 doses of NAT pembro (200 mg) → 15 doses of Adj pembro	21% (NAT → Adj arm)	N/A	<80% at 24 months (Adj arm) >80% at 24 months (NAT → Adj arm)

Abbreviations: Adj, adjutant; dab, dabrafenib; ipi, ipilimumab; N, number of enrolled patients; N/A, not available; NAT, neoadjuvant therapy; nivo, nivolumab; OS, overall survival; pembro, pembrolizumab; rela, relatlimab; RFS, relapse‐free survival; tra, trametinib.

## 6 METASTASECTOMY

In patients with melanoma with resectable distant metastases, such as oligometastasis, the role of metastasectomy is controversial in the era of novel, effective therapeutic agents. Before the era of novel agents, several reports showed that metastectomy prolongs favorable prognosis.^99^, ^100^, ^101^, ^102^ A meta‐analysis^103^ of 31 282 patients (curative metastasectomy, *n* = 9958; non‐curative metastasectomy, *n* = 21 324) with distant metastatic disease revealed that curative metastasectomy was significantly associated with a lower risk of death (HR 0.42, *P* < 0.001). Patients with single‐organ metastases had longer OS than those with multiple metastases and metastatic organ sites. Although the considerable selection bias for curative metastasectomy reduced the evidence strength in this meta‐analysis, the authors concluded that curative metastasectomy should still be a part of the multimodality treatment of stage IV melanoma, such as resectable oligometastatic disease, if technically feasible.^103^

Another study reported the role of metastasectomy in combination with immune checkpoint inhibitors.^104^ In this study, 52 patients with oligometastasis underwent metastasectomy at least 6 months after initiating immune checkpoint inhibitors. The result showed no evidence of disease or non‐progressive residual disease, which resulted in a 3‐year PFS of 31% and a 5‐year MSS of 60%. Stratified by patterns of failure, patients with progression of established tumors had a 3‐year PFS of 70%; in contrast, those with new metastases had a 3‐year PFS of 6% (*P* = 0.001). Five‐year MSS in patients with the progression of established tumors and those with new metastases after metastasectomy were 93% and 31%, respectively (*P* = 0.046). These data indicate that metastasectomy for oligometastasis after immune checkpoint inhibitor treatment may achieve sustained PFS in certain patients. However, we must consider that this study also has a selection bias.

## 7 CONCLUSIONS

Compared with the past standard of care regarding surgery for melanoma, the furture standard of care for surgery will be less invasive, accompanied by the development of novel therapeutic agents for adjuvant or neoadjuvant use and verification of the correlation between surgical procedures, including excision margins for the primary tumor and the extent of CLND, and patients' complications and prognosis.

## CONFLICT OF INTEREST STATEMENT

S.K. has no conflicts of interest to disclose. T.I. received institutional research funding from Kakenhi, Maruho, Taiho, Daiichi Sankyo, Torii, and Sun Pharma, and has received honoraria from BMS, MSD, and Ono Pharma. Y.N. receives institutional research funding from Torii and has served as a consultant or/and has received honoraria from Alexion Pharma, Bristol‐Myers Squibb (BMS), Kyowa Kirin, Leo Pharma, Maruho, Merck Sharp & Dohme (MSD), Novartis, Ono Pharma, Sanofi, Sun Pharma, Tanabe‐Mitsubishi Pharma, and Torii.

## Data Availability

The raw data supporting the conclusions of this article will be made available by the authors, without undue reservation.
